# Synthetic inhibition of SREBP2 and the mevalonate pathway blocks rhabdomyosarcoma tumor growth *in vitro* and *in vivo* and promotes chemosensitization

**DOI:** 10.1016/j.molmet.2024.102085

**Published:** 2024-12-18

**Authors:** Silvia Codenotti, Michela Asperti, Maura Poli, Luisa Lorenzi, Alberto Pietrantoni, Matteo Cassandri, Francesco Marampon, Alessandro Fanzani

**Affiliations:** 1 Department of Molecular and Translational Medicine, University of Brescia, Brescia, Italy; 2 ASST Spedali Civili di Brescia, 25123, Brescia, Italy; 3 Department of Radiological Sciences, Oncology and Anatomic Pathology, “Sapienza” University of Rome, 00161, Rome, Italy; 4 Department of Pediatrics, “Sapienza” University of Rome, Rome, Italy

**Keywords:** Cholesterol, Chemoresistance, Mevalonate pathway, Oxidative stress, Rhabdomyosarcoma

## Abstract

**Objective:**

The aim of the present study was to investigate the effects of targeting the mevalonate pathway (MVP) in rhabdomyosarcoma (RMS), a soft tissue tumor with a prevalence in young people.

**Methods:**

*In silico* analyses of RNA datasets were performed to correlate MVP with RMS patient survival. The sensitivity of RMS cell lines to MVP inhibitors was assessed *in vitro* by analysis of cell growth (crystal violet and clonogenic assays), cell migration (wound healing assay), cell survival (neutral red assay), and oxidative stress (ROS assay). The effects of MVP inhibitors were tested *in vivo* by analyzing RMS xenografts grown in NOD/SCID mice. Quantification of protein targets was performed using immunoblotting or immunohistochemistry analyses.

**Results:**

By analyzing RNA datasets from rhabdomyosarcoma (RMS), a soft tissue tumor with a prevalence in young people, we found *In silico* analysis showed upregulation of sterol regulatory element-binding protein 2 (SREBP2) and mevalonate pathway (MVP) genes, including 3-Hydroxy-3-Methylglutaryl-CoA Reductase (HMGCR), farnesyl-diphosphate synthase (FDPS), squalene epoxidase (SQLE), which correlated with worse overall patient survival and predicted statin sensitivity. Targeting of MVP in human RD and RH30 lines by inhibitors of SREBP2 (fatostatin), HMGCR (lovastatin and simvastatin), and FDPS (zoledronic acid) resulted in In impaired cell growth, migration, and viability, and increased oxidative cell death in combination with actinomycin D. Conversely, cholesterol (CHO) supplementation enhanced cell growth and migration. human RD and RH30 lines, treatment with 0.01–1 μM doses of fatostatin (SREBP2 inhibitor), lovastatin and simvastatin (HMGCR inhibitors), and zoledronic acid (FDPS inhibitor) impaired cell growth and migration, which were conversely stimulated by 50–100 μM cholesterol (CHO) supplementation. Treatment of RMS lines with higher doses of SREBP2 and MVP inhibitors (5–50 μM) promoted oxidative cell death and chemosensitization in combination with actinomycin D. Administration of lFatostatin and lovastatin or fatostatin to RD and RH30 cells produced produced a rapid attenuation of Erk1/2 and Akt1 phosphorylation signaling in RMS lines, and oral administration of lovastatin reduced tumor mass detectable after 4 h of treatment. Furthermore, tumor mass growth of xenografted RD cells in NOD/SCID mice was reduced by oral administration of lovastatin. LastlyFinally, we found we found that the forced Akt1 activation in RD cells was sufficient to drive SREBP2, HMGCR and SQLE protein expression, and enhance cell death promoting increased susceptibility to MVP inhibitors.

**Conclusions:**

Taken together, these data suggest that the axis formed by Akt1, SREBP2 and MVP axis is critical for RMS tumor growth, migration, and oxidative stress protection mainly primarily through the maintenancemaintaining adequate CHO levels that enable of proper intracellular signaling CHO levels that ensure proper intracellular signaling. Therefore, targeting stimulating CHO levels depletion by via SREBP2 and MVP inhibition may represent a viable option to improve the combination therapy protocol, especially in pAkt1-positive RMS.

## Highlights

• Upregulation of SREBP2 and the mevalonate pathway predicts worse survival for patients with rhabdomyosarcoma.
• Cholesterol levels influence cell growth, migration, and chemoresistance.
• Cholesterol depletion reduces Erk and Akt signaling.
• Lovastatin administration reduces tumor xenograft growth in nude mice.
• The Akt pathway drives increased expression of SREBP2 and the mevalonate pathway genes.

## 1 Introduction

RMSs are high-grade malignant tumors with myogenic propensity [1,2]. Even though RMS is rare in adults, it accounts for approximately half of the soft tissue sarcomas in the pediatric age group that includes children, adolescents and young adults. The World Health Organization recognizes three RMS histotypes that arise in young people, i.e. embryonal, alveolar and spindle cell sclerosing RMS [3]. The embryonal and alveolar represent the most frequent cases and have been respectively renamed fusion-negative (FNRMS) and fusion-positive (FPRMS) depending on the absence or presence of recurrent chromosomal translocations involving chromosomes 2 or 1 and chromosome 13, that result in expression of the fusion oncoproteins PAX3-FOXO1 or PAX7-FOXO1 [4]. Spindle cell sclerosing RMS is a rare type characterized by fascicular spindle cell morphology and associated with various non-PAX fusion genes [5,6]. Multimodal treatment requires surgery, radiotherapy, and chemotherapy based on VAI/VAC cocktails (vincristine, actinomycin D, ifosfamide/cyclophosphamide) [7]. Over 90% of patients with low risk localized disease are successfully treated, while overall survival rates for patients with metastatic or recurrent disease remain low [8,9]. RAS and phosphoinositide 3-kinase (PI3K)/Akt activation are considered among the most important factors associated with poor outcomes in RMS [[10], [11], [12]]. The PI3K-Akt pathway is the most activated signaling in human tumors [13,14] and high Akt1 phosphorylation at both Ser473 and Thr308 predicts poor overall survival in RMS patients [15,16]. Akt signaling regulates lipid metabolism through activation of the SREBP family of transcription factors (SREBP1a, SREBP1c, and SREBP2), which induce the expression of nearly all enzymes of fatty acid and sterol synthesis [17]. SREBP2 is mainly involved in CHO biosynthesis and homeostasis given the ability to activate the transcription of MVP genes, such as HMGCR, mevalonate kinase (MVK) and others [18]. Dysregulated MVP contributes to tumor progression and chemotherapy resistance by increasing the levels of several important lipid compounds, including CHO, isoprenoid lipid anchors such as farnesyl pyrophosphate (FPP) and geranylgeranyl pyrophosphate (GGPP), and non-steroidal isoprenoids such as heme A of cytochrome c oxidase, dolichol, and ubiquinone [19]. CHO is used as precursor for the synthesis of steroid hormones, vitamin D, and bile acids, as well as for the maintenance of the cell membrane and the formation of lipid rafts that facilitate transport, signal transduction, and cell polarization [20]. FPP and GGPP are required for post-translational prenylation of many oncogenic proteins, including some RAS family members [21]. MVP pathway also promotes the production of ubiquinone (also known as coenzyme Q10), thereby supporting mitochondrial electron transport [22]. For these reasons, targeting MVP may represent an option for cancer prevention and therapy [[23], [24], [25], [26]]. To date, inhibition of HMGCR by statins has been shown to promote cell apoptosis in RMS [[27], [28], [29], [30], [31]], as well as inhibition of RAS farnesylation by tipifarnib, a potent and selective farnesyltransferase (FTase) inhibitor, was sufficient to produce tumor growth inhibition exclusively in HRAS-mutant RMS xenografts [32]. In this work, using a multifaceted *in silico*, *in vitro* and *in vivo* approach, we investigated the effects of synthetic targeting of SREBP2 and MVP in human RMS cell lines.

## 2 Materials and Methods

### 2.1 *In silico* analysis

Human dataset (GSE108022) is composed of 66 FNRMS, 35 FPRMS, and 3 skeletal muscle samples, whereas mouse dataset (GSE22520) of 7 FNRMS, 11 FPRMS, and 5 skeletal muscle samples. Correlation analysis between MVP genes and tumor grade and between gene expression was performed using a cohort of 101 human RMS patients with clinical informations available at https://www.ebi.ac.uk/biostudies/arrayexpress/studies/E-TABM-1202. Kaplan Meier curve was obtained using R2 platform https://hgserver1.amc.nl/. The DepMap (https://depmap.org/portal/) is a publicly available dataset of more than 1000 cancer cell lines, including 13 RMS cell lines, in which genes have been knocked out by CRISPR/Cas9 technology (www.depmap.org/). Perturbation gene effects were reported as Chronos score. Data were analyzed and plotted as scatter plot using GraphPad Prism 8. Using DepMap we analyzed the PRIMS portal in which 4,518 compounds were tested in 578 cell lines [33]. Across different cancer subtypes, RMS lines reached a score < −3.

### 2.2 Antibodies

Primary antibodies were used for total and phosphorylated Erk1/2 (Thr202/Tyr204) (Santa Cruz Biotechnology, Dallas, TX, USA), total and phosphorylated Akt1 (Ser473), Caveolin-1 (Cav-1), phosphorylated p70 (Thr389), SREBP2, HMGCR, SQLE, (Immunological Sciences, Rome, Italy), and α-Tubulin (Sigma Aldrich, Milan, Italy). The secondary antibodies were anti-mouse and anti-rabbit (Immunological Sciences, Rome, Italy).

### 2.3 Cell culture

Human RD and RH30 cell lines were purchased from the European Collection of Cell Cultures (ECACC, Salisbury, UK). Stable myrAkt1 clones were established in our previous studies [30]. All cell lines were routinely maintained in a humidified incubator at 37 °C, 5% CO_2_ in high-glucose Dulbecco’s Modified Eagle’s Medium (DMEM) (Euroclone, Milan, Italy) supplemented with 10% fetal bovine serum (FBS) (Euroclone, Milan, Italy), 100 mg/ml penicillin/streptomycin antibiotics (Sigma Aldrich, Milan, Italy) and 1 mM l-glutamine (Sigma Aldrich, Milan, Italy).

### 2.4 Drug treatments

Fatostatin, lovastatin, simvastatin, zoledronic acid, mevalonic acid (MVA), LY294002, and actinomycin D were dissolved in dimethyl sulfoxide (DMSO) vehicle. Water-soluble CHO, glutathione (GSH) and N-acetylcysteine (NAC) were dissolved in deionized water. All compounds were from Sigma Aldrich (Milan, Italy).

### 2.5 Crystal violet assay

Cells were seeded in 96-well plates (1.5 × 10^3^) and after 24 h were treated with the indicated compounds. After 48 h, cells were fixed with 3% paraformaldehyde (PFA)/phosphate buffer solution (PBS) solution (20 min, 4 °C) and stained with crystal violet solution (0.2% crystal violet/20% methanol/PBS) (10 min, room temperature (RT)). Cells were washed and resuspended in 1% sodium dodecyl sulfate (SDS)/PBS solution. Plates were shaken until complete dissolution was achieved and then absorbance was measured by reading the plate at 595 nm emission wavelength.

### 2.6 Clonogenic assay

Cells were plated into 6-well plates in triplicate at low cell density (1000 cells/well) and treated with the indicated compounds for 48 h. The medium containing the drugs was then removed and replaced with fresh medium. After 10 days, colonies were fixed with 3% PFA/PBS solution (20 min, 4 °C) and stained with crystal violet solution (0.2% crystal violet/20% methanol/PBS) (10 min, RT). Wells were washed and pictures of colonies were taken. Then, the dye was solubilized in 1% SDS/PBS solution. Plates were shaken until complete dissolution was achieved, and then absorbance was measured by reading the plate at 595 nm emission wavelength.

### 2.7 Wound healing assay

Cells seeded in 6-well plates (2 × 10^5^) formed confluent monolayers that were pre-treated for 2 h with the indicated compounds and then wounded by scraping the cells with a 200 μl-sterile micropipette tip. After 24 h, cells were fixed with 3% PFA/PBS solution (20 min, 4 °C) and stained with crystal violet solution (0.2% crystal violet/20% methanol/PBS) (10 min, RT). Images of wound healing were acquired at different time-points by an inverted light microscope (Olympus IX50; Olympus, Tokyo, Japan) using cellSens Software (Olympus, Tokyo, Japan). The extent of wound repair was quantified by measuring the healed area using ImageJ software. Results were presented as percentage of repaired area with respect to time 0 h.

### 2.8 Neutral red assay

Cells seeded in 96-well plates (1.5 × 10^3^) were left for 24 h in complete medium before receiving treatments. After 48 h, cells were incubated with neutral red solution (40 μg/ml) dissolved in DMEM with 5% FBS (2 h, 37 °C) before washing with neutral red destaining solution (50% ethanol/1% acetic glacial acid/49% deionized water). Plates were shaken until complete dissolution was achieved, and then absorbance was measured by reading the plate at 540 nm emission wavelength.

### 2.9 Oxidative stress analysis

The production of reactive oxygen species (ROS) was quantified using the fluorescent probe CM-H2DCFDA (ThermoFisher Scientific, Milan, Italy), according to the manufacturer’s instructions. Cells were plated in black 96-well plates (2.5 × 10^3^) and after 24 h were treated with the indicated compounds. After 24 h, the supernatant was removed, and cells were incubated with medium containing 1 μM probe (30 min, 37 °C) protected from light. The medium containing the probe was then removed and replaced with fresh medium. The fluorescence was monitored using the EnSight Multimode plate reader (PerkinElmer, Waltham, MA, USA) at 492 nm excitation/517 nm emission wavelengths. Cells were then fixed in 3% PFA/PBS solution (20 min, 4 °C) and processed for the crystal violet assay used as normalization. Results were expressed as the ratio of fluorescence/crystal violet absorbance and reported as the fold change, setting control to 1.

### 2.10 Immunoblotting

Cells were lysed in RIPA buffer, sonicated and centrifuged (10,000×*g*, 10 min, 4 °C). Protein concentration was determined with the Bradford reagent and 40 μg of protein were separated by SDS-PAGE, transferred onto polyvinylidine (PVDF) membranes and blocked with Tris buffered saline (TBS) with 1% Tween-20 (TBS-T) and 5% milk (15 min, RT) prior to incubation with primary antibody (overnight, 4 °C). After 1 h with HRP-conjugated secondary antibody, membranes were TBS-T washed and the resulting immunocomplexes were visualized using enhanced chemiluminescence reagent (GeneSpin, Milan, Italy). Band densitometry was calculated using the Gel Pro Analyzer 4 software (MediaCybernetics Inc., Rockville, MD, USA) and expressed by arbitrary density units.

### 2.11 *In vivo* studies

Tumor xenografts were established by subcutaneous flank injections of 5 × 10^6^ human RD cells (resuspended in 100 μl PBS) into randomly selected 7-week-old male NOD/SCID mice (Envigo, Huntingdon, UK). After 14 days, mice randomly divided into two groups were treated with lovastatin (10 mg/kg) or vehicle (100 μl PBS) via oral gavage 3 times/week for 3 weeks. Tumor growth was measured by a microcaliper twice a week and tumor volume (V) was calculated as follows: *V* = (length x width 2) x 0.5. On day 21, mice were sacrificed, and tumors were removed for further analysis. The study was approved by the Institutional Animal Care and Use Committee of the University of Brescia, Italy.

### 2.12 Immunohistochemistry

Formalin-fixed, paraffin-embedded tumor sections (2 mm) were deparaffinized in xylene and rehydrated through a 100–95% ethanol gradient. Sections were stained with Hematoxylin and Eosin followed by a series of dehydration steps via ethanol washes, cleared with xylene and mounted using Safemount (Bio Optica, Milan, Italy). Heat induced epitope retrieval was done with EDTA buffer pH 8.0 (1 h, 98 °C) and 0.3% H_2_O_2_ was used to block endogenous peroxidases. Sections were then washed in TBS (pH 7.4) and incubated with the specific primary antibody diluted in TBS with 1% bovine serum albumin (BSA) (1 h, RT). Protein signal was revealed by ChemMATE EnVision HRP Labelled Polymer system (DAKO, Glostrup, Denmark) followed by diaminobenzydine as chromogen and Hematoxylin as counterstain. After repeating a series of dehydration steps via ethanol washes, sections were cleared with xylene and mounted using Safemount (Bio Optica, Milan, Italy). Images were acquired by an inverted light microscope (Olympus IX50; Olympus, Tokyo, Japan) using cellSens Software (Olympus, Tokyo, Japan).

### 2.13 Statistical analysis

Statistical significance was assessed by Unpaired Student’s t-test and One-Way ANOVA test, using GraphPad Prism 8 software (GraphPad Software, San Diego, CA, USA). Statements of significance were based on a *p*-value of less than 0.05.

## 3 Results

### 3.1 *In silico* analysis shows that MVP upregulation is associated with worse survival in RMS patients

**Figure 1:**
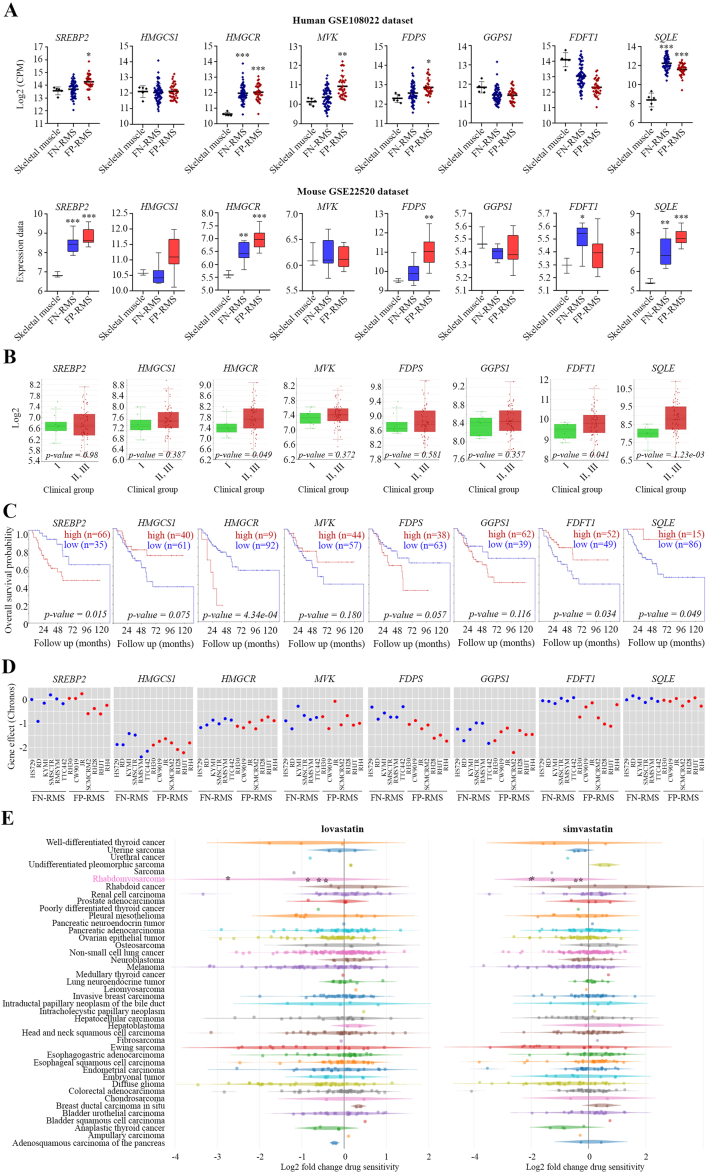
*In silico* analysis. A) The analysis of SREBP2 and MVP gene expression was performed using human and mouse RMS tumor datasets (see Materials and Methods for the composition of the datasets). B) Correlation analysis between gene expression and tumor grade in RMS patients. Clinical group I (*n* = 8), clinical group II, III (*n* = 64). C) Kaplan Meier curves showing overall survival of RMS patients in relation to gene expression. D) Sensitivity to SREBP2 and MVP gene deletion in RMS lines. On the y-axis, the effect of gene deletion (Chronos) is significant when it is less than −0.5. E) Statin sensitivity across several cancer lines. In the graph, the various tumor lines are shown on the y-axis, while the sensitivity to the drugs lovastatin and simvastatin on the x-axis increases towards the left. RMS lines are highlighted in pink. (For interpretation of the references to colour in this figure legend, the reader is referred to the Web version of this article.)

Using RNA datasets from human and mouse RMS, we assessed the expression levels of SREBP2 and of MVP genes. In both datasets, we found upregulation of SREBP2, HMGCR, FDPS, and SQLE compared with skeletal muscle samples (Figure 1A). By analyzing the clinical information of 101 RMS patients, we observed that the upregulation of MVP genes, especially HMGCR, farnesyl-diphosphate farnesyltransferase 1 (FDFT1), and SQLE, was correlated with the advanced tumor grade (Figure 1B). Furthermore, elevated levels of SREBP2 and HMGCR were significantly associated with worse overall survival of RMS patients, as revealed by Kaplan Meier curves (Figure 1C). Using the DepMap database, we found that deletion of almost all MVP genes was detrimental to survival in several FPRMS (*n* = 7) and FNRMS (*n* = 6) cell lines (Figure 1D). Additionally, using the PRISM screening portal [33], pronounced sensitivity to the HMGCR inhibitors lovastatin and simvastatin was found in some tumors, including RMS, melanoma, Ewing Sarcoma, hepatocellular carcinoma, and diffuse glioma (Figure 1E). Taken together, these data suggest a potential role for SREBP2 and MVP in the progression of RMS.

### 3.2 Cell growth and migration of RMS lines are strongly impaired by SREBP2 and MVP inhibitors, but increased by CHO supplementation

**Figure 2:**
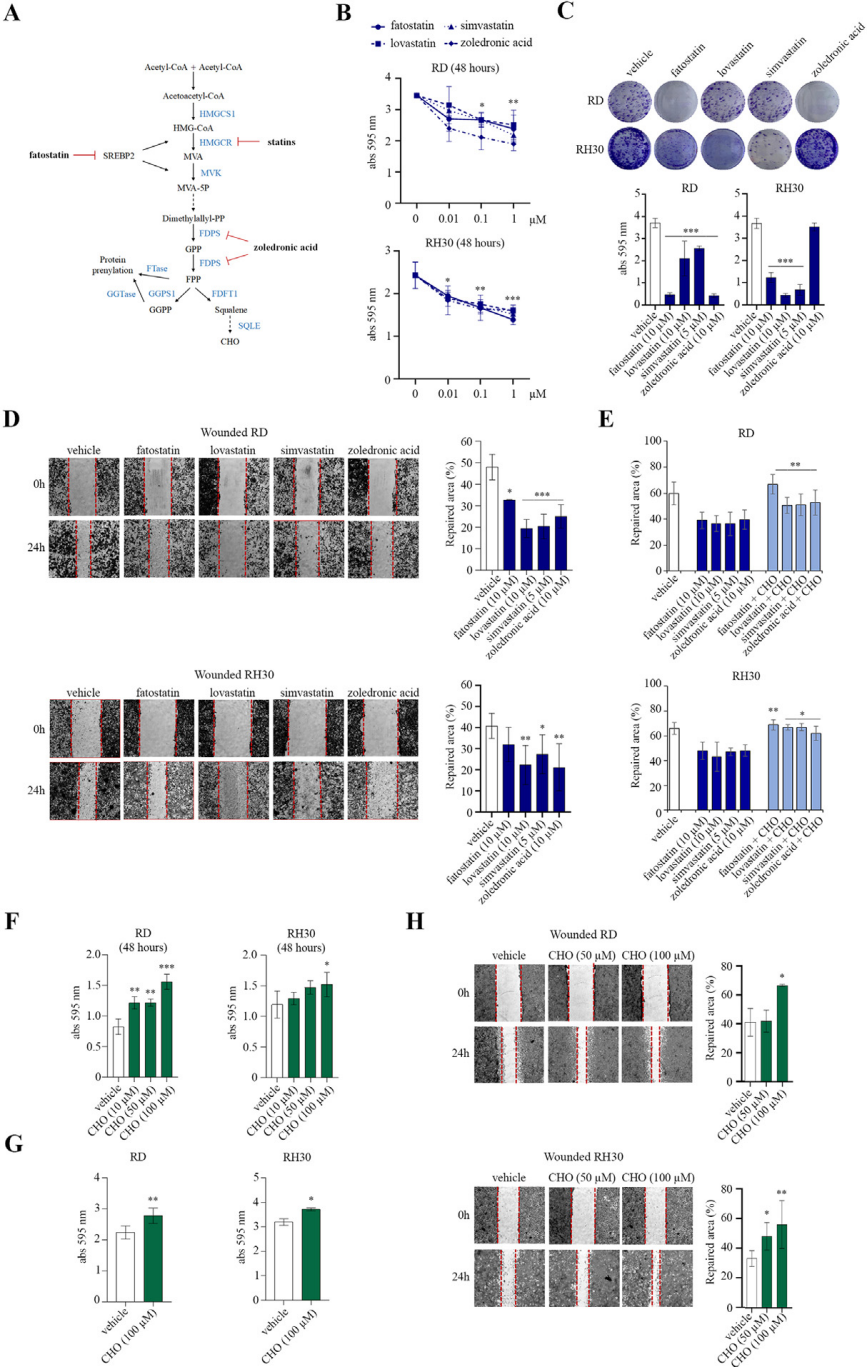
Analysis of RMS cell growth and migration in the presence of SREBP2 and MVP inhibitors and CHO supplementation. A) MVP begins with the formation of HMG-CoA from three molecules of acetyl-CoA catalyzed by the enzyme HMG-CoA synthase (HMGCS). HMG-CoA is then converted to MVA by HMGCR, the target of statins. MVA is phosphorylated by MVK and converted into two five-carbon building blocks called isopentenyl pyrophosphate and dimethylallyl pyrophosphate (dimethylallyl-PP). This step is crucial for the biosynthesis of FPP by a cascade of several synthases, including FDPS. FPP can then be converted to squalene by FDFT1 and, subsequently, to CHO by further enzymes such as squalene synthase and SQLE. Alternatively, FPP and GGPP can be used for protein prenylation by FTase and geranylgeranyl transferase (GGTase). The inhibitors used to block the pathway are indicated in bold. The gene targets of SREBP2 are HMGCR and MVK. B) Cell proliferation of RD and RH30 lines, treated with inhibitors or vehicle, was assessed after 48 h by crystal violet incorporation (*n* = 3). Data are mean ± SEM, ∗*p*-value <0.05, ∗∗*p*-value <0.001, ∗∗∗*p*-value <0.0001; one-way ANOVA test. C) Clonogenic capacity of RMS lines, treated with inhibitors or vehicle, was assessed after 10 days by colony visualization after crystal violet incorporation and relative spectrophotometric quantification (*n* = 3). Data are mean ± SEM, ∗∗∗*p*-value <0.0001; one-way ANOVA test. D) Wound repair in RMS lines pretreated for 2 h with SREBP2 and MVP inhibitors or vehicle, as detected after 24 h (*n* = 3). In the images, wound edges at different time points are identified as red dashed lines. The percentage of repaired area is reported in the graphs. Data are mean ± SEM, ∗*p*-value <0.05, ∗∗*p*-value <0.001, ∗∗∗*p*-value <0.0001; one-way ANOVA test. E) The graphs report the percentage of repaired area, detected after 24 h, relative to the wound healing assay on RMS lines treated with SREBP2 and MVP inhibitors with or without 100 μM CHO supplementation (*n* = 3). Data are mean ± SEM, ∗*p*-value <0.05, ∗∗*p*-value <0.001; unpaired Student’s t-test. F) Cell proliferation of RMS lines treated with CHO was assessed after 48 h by crystal violet incorporation (*n* = 3). Data are mean ± SEM, ∗*p*-value <0.05, ∗∗*p*-value <0.001, ∗∗∗*p*-value <0.0001; one-way ANOVA test. G) Clonogenic capacity of RMS lines treated with CHO or vehicle was assessed after 10 days by colony visualization after crystal violet incorporation and relative spectrophotometric quantification (*n* = 2). Data are mean ± SEM, ∗*p*-value <0.05, ∗∗*p*-value <0.001; unpaired Student’s t-test. H) Wound repair in RMS lines pretreated for 2 h with CHO or vehicle, as detected after 24 h (*n* = 3). The wound edges at different time points are identified as red dashed lines. The percentage of the area repaired is reported in the graphs. Data are mean ± SEM, ∗*p*-value <0.05, ∗∗*p*-value <0.001; one-way ANOVA test. (For interpretation of the references to colour in this figure legend, the reader is referred to the Web version of this article.)

We treated RD and RH30 lines with 0.01–1 μM doses of SREBP2 and MVP inhibitors, including fatostatin, lovastatin and simvastatin, and zoledronic acid, as schematically illustrated in Figure 2A. After 48 h, cell proliferation of RMS lines was reduced with all compounds compared to untreated cells, as shown by the crystal violet assay (Figure 2B). By a clonogenic assay, these compounds also showed inhibitory effects on long term cell growth (Figure 2C). Zoledronic acid reduced the colony growth of RD, but not RH30 cells (Figure 2C). The effects of MVP inhibitors on cell migration were then tested by performing a wound healing assay. All inhibitors significantly reduced the repair of the damaged area after 24 h in both RMS lines, as visible from the images and relative quantifications (Figure 2D). Concomitant treatment with MVP inhibitors and CHO significantly promoted a recovery of cell migration (Figure 2E). Furthermore, we found that CHO supplementation enhanced cell proliferation, growth, and migration in both RD and RH30 lines, as assessed by crystal violet, clonogenic, and wound healing assays, respectively (Figure 2F–H). Overall, these data suggest that MVP, through increasing intracellular CHO levels, influences the growth and migration abilities of RMS lines.

### 3.3 SREBP2 and MVP inhibitors promote lethal oxidative stress and chemosensitization

**Figure 3:**
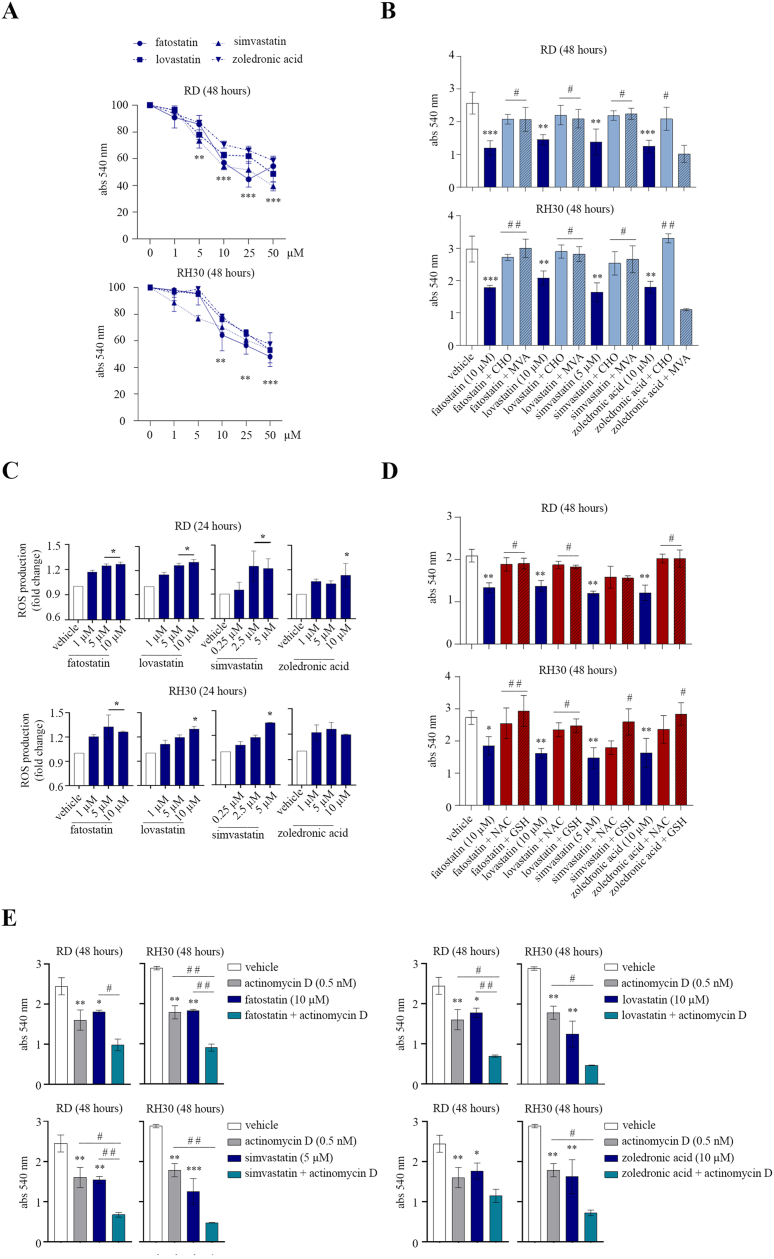
Effects of SREBP2 and MVP inhibitors on cell viability and oxidative stress levels. A) Cell viability of RMS lines treated with the indicated inhibitors or vehicle was assessed after 48 h by neutral red assay (*n* = 3). Data are mean ± SEM, ∗∗*p*-value <0.001; ∗∗∗*p*-value <0.0001; one-way ANOVA test. B) RMS cell viability was assessed after cotreatment with the indicated inhibitors and 100 μM CHO or 1 mM MVA by neutral red assay after 48 h. Data are mean ± SEM, ∗∗*p*-value <0.001; ∗∗∗*p*-value <0.0001; unpaired Student’s t-test vs. vehicle-treated cells. #*p*-value <0.05; ##*p*-value <0.001; one-way ANOVA test vs. single treatment. C) Fluorometric ROS quantification in RMS lines, pre-incubated with the CM-H2DCFDA probe, was assessed after treatment with the indicated inhibitors or vehicle for 24 h (*n* = 2). Data are mean ± SEM, ∗*p*-value <0.05; one-way ANOVA test. D) Cell viability of RMS lines treated with the indicated inhibitors, in the presence or absence of a 24 h-long pretreatment with 10 mM NAC or GSH, was assessed by neutral red assay after 48 h. Data are mean ± SEM, ∗*p*-value <0.05; ∗∗*p*-value <0.001; unpaired Student’s t-test vs. vehicle-treated cells. #*p*-value <0.05; ##*p*-value <0.001; one-way ANOVA test vs. single treatment. E) Cell viability of RMS lines, treated with the indicated inhibitors alone or in combination with actinomycin D, was assessed by neutral red assay after 48 h (*n* = 2). Data are mean ± SEM, ∗*p*-value <0.05; ∗∗*p*-value <0.001; ∗∗∗*p*-value <0.0001; one-way ANOVA test vs. vehicle-treated cells. #*p*-value <0.05; ##*p*-value <0.001; one-way ANOVA test vs. single treatment. (For interpretation of the references to colour in this figure legend, the reader is referred to the Web version of this article.)

By neutral red assay we found that treatment of RMS lines with 5–50 μM doses of SREBP2 and MVP inhibitors promoted a significant reduction (up to 40%) in cell viability, as detected after 48 h (Figure 3A). Cell death induced by MVP inhibitors was reversed by CHO supplementation but not by MVA, as assessed by the neutral red assay (Figure 3B), confirming an important role of CHO in protecting against cell death induced by SREBP2 and MVP inhibitors. Since statins are known to promote oxidative stress in several tumors [34], including RMS [31], the RMS lines were incubated with a specific probe sensitive to oxidative levels, named CM-H2DCFDA, before treatment with MVP inhibitors. We observed a dose-dependent increase in intracellular ROS levels within 24 h of treatment (Figure 3C), and the cell death induced by SREBP2 and MVP inhibitors was significantly reduced by co-treatment with antioxidant scavengers, such as NAC and GSH, as shown by the neutral red assay (Figure 3D). Finally, we found that the combination of SREBP2 and MVP inhibitors with actinomycin D was particularly effective in reducing cell viability of RMS lines, as detected after 48 h of treatment (Figure 3E). Overall, these data suggest that SREBP2 and MVP inhibitors cause oxidative cell death and promote chemosensitization towards actinomycin D.

### 3.4 MVP inhibition produces rapid downregulation of Erk and Akt phosphorylation *in vitro* and reduction of tumor growth *in vivo*

**Figure 4:**
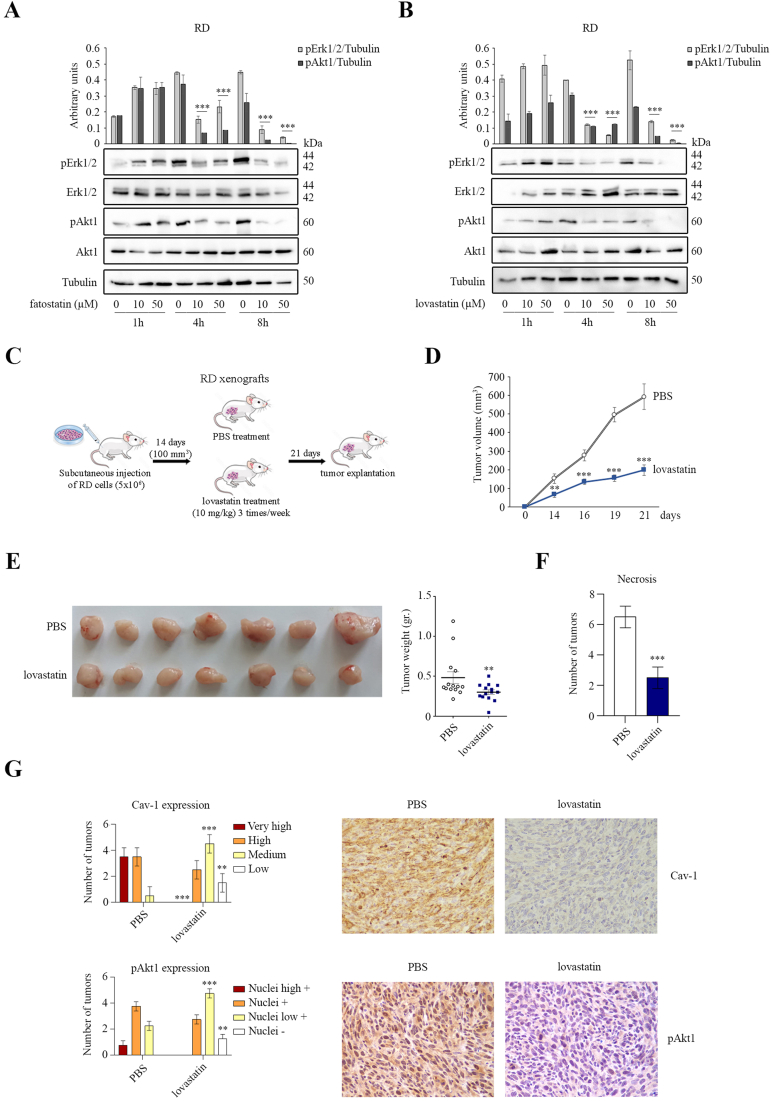
Analysis of intracellular signaling and *in vivo* tumor growth of RD line treated with fatostatin and lovastatin. A-B) Immunoblotting was performed to detect total and phosphorylated Erk1/2 and Akt1 proteins in RD cells treated with the indicated drugs. Tubulin serves as loading control and protein quantification is expressed by arbitrary density units (*n* = 3). Data are Mean ± SEM, ∗∗∗*p*-value<0.0001; One-Way ANOVA test. C) Schematic representation of experimental protocol used for the *in vivo* tumor growth. After subcutaneous injection of human RD cells (5 × 10^6^ cells) into NOD/SCID mice (*n* = 14), the tumor xenografts grew for 14 days to an average size of 100 mm^2^. Subsequently, one group of mice was treated with lovastatin (*n* = 7) and one with vehicle (*n* = 7) 3 times/week for 21 days. The experiment was repeated twice. D) The size of tumors was estimated four times during subcutaneous growth (*n* = 28). Data are Mean ± SEM, ∗∗*p*-value <0.001; ∗∗∗*p*-value <0.0001; unpaired Student’s t-test. E) Representative images of tumors were taken after 21 days. The weight of collected tumors was reported in the graph (*n* = 28). Data are Mean ± SEM, ∗∗∗*p*-value <0.0001; unpaired Student’s t-test. F) The number of tumors showing necrotic areas was quantified (*n* = 28). Data are Mean ± SEM, ∗∗∗*p*-value<0.0001; unpaired Student’s t-test. G) Images (200X) showing immunohistochemical staining of Cav-1 and pAkt1 in tumor samples. The number of cases was classified based on signal intensity and quantified as reported in the graphs. Data are Mean ± SEM, ∗∗*p*-value <0.001; ∗∗∗*p*-value <0.0001; unpaired Student’s t-test.

Since CHO is a key component of lipid rafts, we wondered whether the inhibition of CHO synthesis could affect intracellular signaling. To address this question, we analyzed the activation of Erk and Akt pathways in RMS lines after treatment with fatostatin and lovastatin. These drugs produced a reduction in phosphorylated Erk1/2 and Akt1 forms after 4 h of treatment in RD cells, as demonstrated by IB analysis (Figure 4A,B), and similar results were obtained in RH30 cells (Supplementary Figs. 1A and B). To assess the effects of MVP inhibition *in vivo*, RD cells were xenografted subcutaneously into NOD/SCID mice (*n* = 28) and allowed to grow for 14 days before mice were administered lovastatin via oral gavage (10 mg/kg) or PBS three times a week (Figure 4C). After two weeks, tumor masses of lovastatin-treated mice were visibly reduced compared to PBS-treated mice (Figure 4D), as confirmed by assessment of mean tumor size (Figure 4D) and weight (Figure 4E) after three weeks. On average, tumors from lovastatin-treated mice reached half the weight of those in PBS-treated mice (Figure 4E). By macroscopic evaluation of paraffin-embedded samples, necrotic areas in lovastatin-treated tumors were significantly reduced compared to PBS-treated tumors (Figure 4F). Furthermore, immunohistochemistry (IHC) analysis on tumor samples showed that the staining for Cav-1, a CHO-binding protein, and pAkt1 (Figure 4F) was completely absent in lovastatin-treated tumors compared to PBS-treated tumors (Figure 4G). Overall, these data clearly indicate a critical role of CHO levels for the correct activation of the Erk and Akt pathways and tumor growth *in vivo*.

### 3.5 In RD cells, the Akt pathway increased the expression of SREBP2 and MVP enzymes and the susceptibility to fatostatin and lovastatin

**Figure 5:**
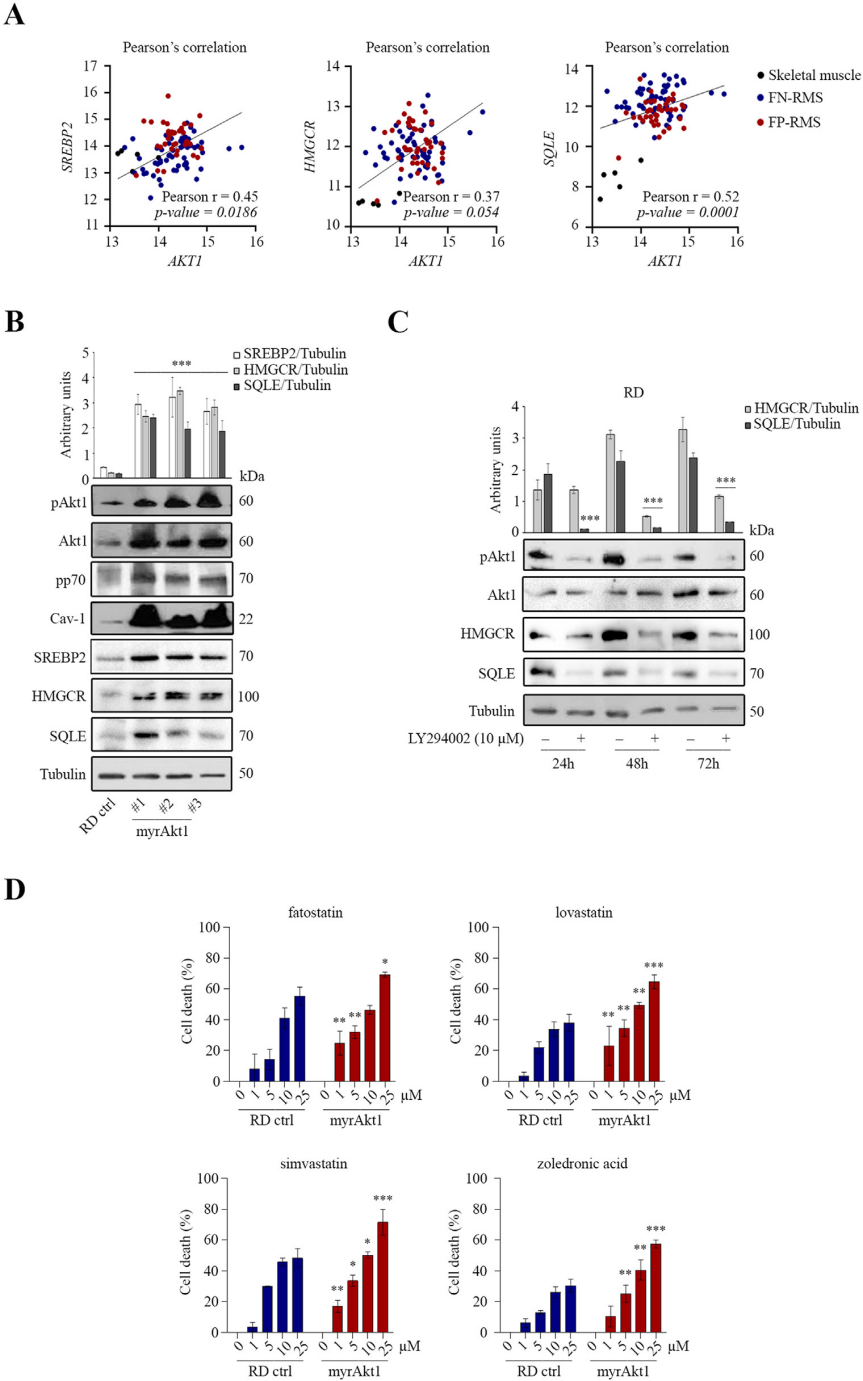
Analysis of Akt1 pathway activation on SREBP2 and MVP enzymes expression. A) The correlation between AKT1 expression and SREBP2, HMGCR and SQLE was calculated by Pearson’s coefficient using the human RNA dataset. B) RD and myrAkt1 cells were left to proliferate for 48 h before collecting protein homogenates for immunoblotting analysis of the indicated proteins (*n* = 2). Data are mean ± SEM, ∗∗∗*p*-value <0.0001; one-way Anova test. C) RD cells were treated with the PI3K inhibitor LY294002 or with vehicle up to 72 h. Immunoblotting analysis of the indicated proteins was performed at different time-points. Tubulin serves as loading control and protein quantification is expressed by arbitrary density units (*n* = 3). Data are Mean ± SEM, ∗∗∗*p*-value<0.0001; One-Way ANOVA test. D) RD and myrAkt1 cells were treated with the indicated inhibitors or with vehicle. Cell viability was assessed by neutral red assay after 48 h and is expressed as percentage of cell death compared to vehicle-treated cells (*n* = 2). Data are mean ± SEM, ∗*p*-value <0.05; ∗∗*p*-value <0.001; ∗∗∗*p*-value <0.0001; unpaired Student’s t-test. (For interpretation of the references to colour in this figure legend, the reader is referred to the Web version of this article.)

The Akt pathway has been described as a master regulator of MVP in several tumors [17,35,36]. Through an in-silico analysis of the human RMS dataset, we observed a significant Pearson's correlation between the expression levels of *AKT1* and *SREBP2*, *HMGCR*, and *SQLE* (Figure 5A). In line with this evidence, increased activation of the Akt/mTOR/p70/Cav-1 pathway, achieved by stable expression of a myristoylated Akt1 form (myrAkt1) in RD cells [30,31], led to increased expression of SREBP2, HMGCR and SQLE proteins compared to control cells (Figure 5B). Conversely, inhibition of the Akt1 pathway by treating RD cells with the PI3K inhibitor (LY294002), caused a downregulation of HMGCR and SQLE protein levels after 24 h (Figure 5C). We found that treatments with 1 μM fatostatin, lovastatin, and simvastatin, promoted a remarkable increase in cell death of myrAkt1 cells compared to control cells, as evaluated by neutral red assay (Figure 5D). These data, highlighting that the Akt1 pathway drives the increase in SREBP2 and MVP expression, suggest that RMS tumors bearing high pAkt1 levels could be sensitive to therapeutic treatments with fatostatin and statins.

## 4 Discussion

Targeting lipid metabolism represents an important anti-cancer strategy [37,38]. The observed correlation between SREBP2 and some MVP genes, such as HMGCR, with tumor grade and worse survival in RMS patients suggests that synthetic targeting of SREBP2 and MVP could be an attractive option to improve the multimodal treatment. SREBP2 is a master regulator of CHO metabolism [39] and is commonly found activated in various cancers [40]. Activated SREBP2, to control CHO biosynthesis and homeostasis, enters the nucleus and binds to SRE, a sterol regulatory element in the promoter region to regulate transcriptional expression of target genes such as HMGCR [41], MVK [18], low-density lipoprotein receptor (LDLR) [42,43], and others. Targeting SREBP2 has been shown to be a promising antitumor therapy in several cancers [40], such as colon [44], prostate and brain tumors [[45], [46], [47]]. Interestingly, fatostatin has been reported to affect Akt signaling in glioblastoma tumors, promoting increased cell death by ferroptosis [48]. Mechanistically, fatostatin has been shown to inhibit tubulin polymerization, activate the spindle assembly checkpoint, and trigger mitotic catastrophe [49]. Dysregulation of MVP promotes transformation [50] and epidemiological studies have confirmed a positive association between high serum CHO levels and cancer risk [51]. Reduction of MVP by HMGCR statin inhibitors, the most widely used drugs worldwide for the treatment of hypercholesterolemia, has been shown to exert antitumor effects through a variety of distinct mechanisms, such as increased oxidative stress and apoptosis [52], induction of cell cycle arrest, and inhibition of cell proliferation and invasion [34]. The data provided in the current work are consistent with an important role for SREBP2 and MVP in supporting RMS cell growth and migration via increased CHO biosynthesis. Additionally, we have observed that dual treatment with SREBP2 or MVP inhibitors and actinomycin D, an oxidative stress inducer used in the VAI/VAC cocktail [53], promotes lethal oxidative stress, suggesting that this combination could be considered for randomized clinical trials. MVP is involved in the synthesis of CHO and many other isoprenoid and non-steroidal intermediates, such as FPP and GGPP, heme A of cytochrome c oxidase, dolichol, and CoQ [19]. Therefore, it is not obvious to determine which MVP intermediate may play a key role in tumor growth. For example, statins were reported to exert anticancer effects by CHO depletion in prostate cancer cells and in medulloblastoma [54,55]. In other works, however, statin-induced apoptosis can be consistently rescued by exogenous GGPP [56] or CoQ [22,57], highlighting that the different MVP intermediates could play a key role depending on tumor type and, especially, tumor stage. Evidence from this study cautiously suggests an important role for CHO in stimulating the growth and migration of RMS lines. Furthermore, we observed that SREBP2 and MVP inhibitors rapidly depressed the Erk and Akt pathways, two critical intracellular signaling with anti-apoptotic activities [58,59]. It is known that the reduction of isoprenoids and CHO levels following MVP inhibition affects cellular signaling with diverse modalities. The isoprenoids FPP and GPP are required for the prenylation of many RAS and RHO families of proteins [32], [60], and thus targeting protein prenylation can result in impaired signal transduction. On the other hand, plasma membrane CHO governs membrane fluidity and receptor organization into caveolar and non-caveolar lipid rafts [61], which function as hubs for signaling proteins [62]. CHO depletion has been shown to alter the function of many receptor and non-receptor signaling molecules, such as EGFR [63], PI3K [64], SRC-family kinases [65], and NOX [66], leading to a reduction in intracellular signaling. In RMS lines, given the pro-tumorigenic effects observed with CHO supplementation and the downregulation of intracellular signaling observed after MVP inhibition, we speculate that CHO abundance plays a crucial role in proper intracellular signaling. The reduction in tumor growth of xenografted RD cells obtained thanks to lovastatin confirms the importance of targeting the MVP in RMS. To our knowledge, this is the first report showing the effect of statin administration on RMS tumor growth *in vivo*. Unlike hydrophilic statins such as pravastatin and rosuvastatin, which mainly suppress CHO biosynthesis by the liver, lovastatin is more bioavailable in the periphery and thus seems to be suitable for targeting MVP in tumor cells [67,68]. Tumor samples from lovastatin-treated mice were characterized by reduced cellular necrosis, the disappearance of Cav-1, a CHO binding protein [69] that exerts a pro-tumorigenic role in RMS [[70], [71], [72], [73]], and by the reduction of pAkt1 [54], [76], [77], indicating that lovastatin-treated tumors were characterized by reduced malignancy. Finally, we demonstrated that Akt1, by increasing the expression of SREBP2, HMGCR and SQLE proteins, is a driver of MVP, as similarly observed in other tumors [74], [75]. In RMS, Akt1 phosphorylation is often found at both Ser473 and Thr308 and predicts poor overall survival in patients [16], and we have recently shown that the Akt1/Src-kinase/Cav-1 signaling axis protects RMS cells from ROS-induced damage via enhanced DNA repair and ROS detoxification [30], [31]. Overall, this suggests that pAkt1-positive RMS tumors may take advantage of the MVP pathway to increase tumor aggressiveness by oxidative stress resistance. In conclusion, these data suggest that SREBP2 and MVP are potential therapeutic targets to counteract tumor growth and promote chemosensitization in RMS.

## CRediT authorship contribution statement

**Silvia Codenotti:** Visualization, Validation, Methodology, Investigation, Formal analysis, Conceptualization. **Michela Asperti:** Visualization, Investigation. **Maura Poli:** Visualization, Investigation. **Luisa Lorenzi:** Visualization, Investigation. **Alberto Pietrantoni:** Visualization, Investigation. **Matteo Cassandri:** Formal analysis. **Francesco Marampon:** Resources, Funding acquisition. **Alessandro Fanzani:** Writing – original draft, Visualization, Supervision, Resources, Funding acquisition, Formal analysis, Conceptualization.

## Declaration of competing interest

The authors declare that they have no known competing financial interests or personal relationships that could have appeared to influence the work reported in this paper.

Alessandro Fanzani on Behalf of all authors

## Figures and Tables

**Figure:**
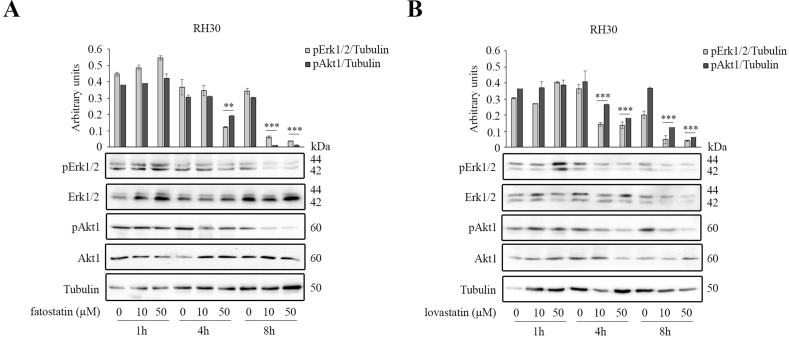
Supplementary Figure 1Effects of lovastatin and fatostatin on intracellular signaling of RH30 line. A-B) Immunoblotting was performed to detect total and phosphorylated Erk1/2 and Akt proteins in response to increasing doses of the indicated drugs. Tubulin serves as loading control and protein quantification is expressed by arbitrary density units (*n* = 3). Data are Mean ± SEM, ∗∗∗*p*-value<0.0001; One-Way ANOVA test.

## Data Availability

Data will be made available on request.
